# Plasmatic Levels of IL-18, IP-10, and Activated CD8^+^ T Cells Are Potential Biomarkers to Identify HIV-1 Elite Controllers With a True Functional Cure Profile

**DOI:** 10.3389/fimmu.2018.01576

**Published:** 2018-07-11

**Authors:** Fernanda H. Côrtes, Hury H. S. de Paula, Gonzalo Bello, Marcelo Ribeiro-Alves, Suwellen S. D. de Azevedo, Diogo G. Caetano, Sylvia L. M. Teixeira, Brenda Hoagland, Beatriz Grinsztejn, Valdilea G. Veloso, Monick L. Guimarães, Mariza G. Morgado

**Affiliations:** ^1^Laboratório de Aids e Imunologia Molecular, Instituto Oswaldo Cruz, FIOCRUZ, Rio de Janeiro, Brazil; ^2^Laboratório de Pesquisa Clínica em DST/Aids, Instituto Nacional de Infectologia Evandro Chagas, FIOCRUZ, Rio de Janeiro, Brazil

**Keywords:** HIV-1, inflammation, immune activation, elite controller, IP-10, IL-18

## Abstract

Elite controllers (ECs) are rare individuals able to naturally control HIV-1 replication below the detection limit of viral load (VL) commercial assays. It is unclear, however, whether ECs might be considered a natural model of a functional cure because some studies have noted CD4^+^ T cell depletion and disease progression associated with abnormally high levels of immune activation and/or inflammation in this group. Here, we propose the use of immunological parameters to identify HIV-1 ECs that could represent the best model of a functional cure. We compared plasma levels of six inflammatory biomarkers (IP-10, IL-18, sCD163, sCD14, CRP, and IL-6) and percentages of activated CD8^+^ T cells (CD38^+^HLA-DR^+^) between 15 ECs [8 with persistent undetectable viremia (persistent elite controllers) and 7 with occasional viral blips (ebbing elite controllers)], 13 viremic controllers (VCs—plasma VL between 51 and 2,000 RNA copies/mL), and 18 HIV-1 infected patients in combined antiretroviral therapy, with suppressed viremia, and 18 HIV-uninfected controls (HIV-neg). The two groups of ECs presented inflammation and activation profiles similar to HIV-neg individuals, and there was no evidence of CD4^+^ T cell decline over time. VCs, by contrast, had higher levels of IL-18, IP-10, and CRP and a lower CD4/CD8 ratio than that of HIV-neg (*P* < 0.05). Plasma levels of IL-18 and IP-10 correlated positively with CD8^+^ T cell activation and negatively with both CD4/CD8 and CD4% in HIV-1 controllers. These results suggest that most ECs, defined using stringent criteria in relation to the cutoff level of viremia (≤50 copies/mL) and a minimum follow-up time of >5 years, show no evidence of persistent inflammation or immune activation. This study further suggests that plasmatic levels of IL-18/IP-10 combined with the frequency of CD8^+^CD38^+^HLA-DR^+^ T cells can be important biomarkers to identify models of a functional cure among HIV-1 ECs.

## Introduction

HIV controllers (HICs) are HIV-1-infected individuals able to control viral replication in the absence of combined antiretroviral therapy (cART) (1). According to the level of control, the HICs are divided into two groups: elite controllers (ECs), individuals able to keep viremia below the limit of detection of viral load (VL) commercial kits (currently, <40–50 RNA copies/mL), and viremic controllers (VCs), individuals able to maintain the plasmatic VL below 2,000 RNA copies/mL (2). Despite the exceptional control of viremia, there are controversies as to whether EC might be considered a natural model of a functional cure.

Some ECs display a progressive loss of CD4^+^ T cell counts and eventually progress to AIDS over time. The disease progression in those individuals appears to be mostly driven by persistent immune activation and inflammation likely associated with residual viremia (3–5). Consistent with this evidence, some studies suggest that the cART might lead to a marked decrease in immune activation and increased CD4^+^ T cell counts in ECs, reducing the risk of non-AIDS-related events (6–8). Conversely, other studies describe that elevated levels of T cell activation and soluble inflammation markers are not associated with a faster rate of CD4^+^ T cell decline in ECs (9, 10) or that ECs maintain absolute CD4^+^ T cell counts and T cell activation levels within the normal range over time (10–12), thus concluding that these individuals may not have benefited from early cART initiation.

These observations confirm that ECs are heterogeneous with regard to both virologic (5, 11, 13, 14) and immunologic (2, 3, 10, 12, 15, 16) features. Such heterogeneity may result from the absence of a standardized classification of ECs mainly in relation to the cutoff level of viremia (≤50–500 copies/mL), minimum follow-up time (1–10 years), and/or presence of occasional blips (17). That heterogeneity may also reflect different underlying mechanisms of natural suppression of viremia across individuals.

The aim of this study was to evaluate different immunologic parameters in a group of ECs with stringent criteria for a definition of both the VL cutoff limit of detection (≤50 copies/mL) and the durability of viral suppression (>5 years) to identify those individuals who could represent the best model of a natural functional cure. To this end, we compared the plasma levels of inflammatory biomarkers and T cell activation between ECs with persistent undetectable viremia, ECs with occasional viral blips, VCs, cART-treated patients with undetectable viremia and HIV-uninfected controls. We also assessed the relationship between CD4^+^ T cell counts, CD4%, CD4/CD8 ratio, levels of CD8^+^ T cell activation, and levels of soluble markers of inflammation among HIV-1 controllers.

## Materials and Methods

### Study Subjects and Ethical Issues

Twenty-eight HIV-1 controllers from the Instituto Nacional de Infectologia Evandro Chagas (INI) were selected for this study and divided into three groups as described previously in Ref. (14): (1) persistent elite controllers (PECs), if 100% of VL measures were below the limit of detection (<50–80 copies/mL) depending on the commercial method available, along with the clinical and laboratory follow-up (*n* = 8); (2) ebbing elite controllers (EECs), if subjects had occasional (<30% of frequency) episodes of transient low-level (51–400 copies/mL) viremia (*n* = 7); and (3) VCs, if most (≥70%) VL determinations were between 51 and 2,000 copies/mL (*n* = 13). Occasional VL measurements above the upper limits were accepted along with the follow-up for the EEC and VC groups. A group of HIV-1 infected individuals on cART with a VL suppressed for at least 2 years (cART; *n* = 18) and a group of HIV-1-uninfected individuals (HIV-neg; *n* = 18) were also included as controls. All participants provided written informed consent, and the ethical committee of Instituto Nacional de Infectologia Evandro Chagas (INI-Fiocruz) approved the study (CAAE 1717.0.000.009-07).

### CD4^+^ T Cell Count and VL Measurement

Absolute CD4^+^ T and CD8^+^ T cell counts were obtained using the Tritest or MultiTest TruCount kit and the MultiSet software on a FACSCalibur flow cytometer (BD Biosciences, San Jose, CA, USA). Plasma VL was measured using the Nuclisens HIV-1 RNA QT assay (Organon Teknika, Durham, NC, USA; limit of detection: 80 copies/mL) from 1999 to 2008, the Versant HIV-1 3.0 RNA assay (bDNA 3.0, Siemens, Tarrytown, NY, USA; limit of detection: 50 copies/mL) from 2008 to 2013, and the Abbott Real Time HIV-1 assay (Abbott Laboratories, Wiesbaden, Germany; limit of detection: 40 copies/mL) since 2013. When available, absolute CD4^+^ T and CD8^+^ T cell counts and VL data were collected from 1997 to 2017, depending on the HIC study entry.

### Markers of T Cell Activation and Inflammation

Cryopreserved PBMCs were thawed in RPMI 1640 medium, GlutaMax supplemented (Gibco, Invitrogen, Carlsbad, CA, USA) containing 10% FBS (R10 medium) (Gibco, Invitrogen, Carlsbad, CA, USA), then washed using R10 medium and incubated overnight at 37°C, 5% CO_2_ and controlled humidity. Afterward, the cells were washed and stained with FVS450 for viability evaluation (BD Biosciences, San Diego, CA, USA) and the following monoclonal antibodies: anti-CD3 APC-H7, anti-CD4 PECF594, anti-CD8 APC, anti-CD38 BB515, and anti-HLA-DR PE (BD Biosciences, San Jose, CA, USA). Then, the cells were washed, fixed with 1% PFA (Sigma, Germany), and acquired using a BD FACSAria IIu Flow Cytometer (BD Biosciences, San Jose, CA, USA). Flow cytometric analysis was performed with Flow Jo v.10.0.7 (Tree Star Inc., Ashland, OR, USA).

Plasmatic levels of IP-10, IL-18, sCD163, sCD14, CRP, and IL-6 were measured using commercial ELISA assays (R&D systems, USA), following the manufacturer’s instructions.

### Statistical Analysis

In the evaluation of the sociodemographic, clinical, and laboratorial features among the different groups of HICs, cART, and HIV-1-uninfected individuals, for continuous numerical variables, Kruskal–Wallis ANOVA by Ranks tests were used for assessing the hypothesis that the different samples in the comparison were drawn from the same distribution or from distributions with the same median. Likewise, for categorical nominal variables, Fisher’s exact tests were used in the evaluation of frequencies among the different groups for assessing the hypothesis of independence between the groups of individuals and these variables. In addition, graphical exploratory analyses were performed for continuous numerical variables for dimension reduction and visualization by multivariate principal component analysis (PCA), and Spearman’s rank correlation coefficient analyses were calculated for these variables. Pairwise comparisons of laboratory variables averaged among groups of interest were performed by contrasts obtained after both bi- and multivariate-linear models fitted by ordinary least square regressions. *P*-values were corrected by the Tukey Honest Significant Difference (HSD) method (18). After all laboratory variable pairwise comparisons, we conducted a type I error adjustment for multiple comparisons following the Holm–Bonferroni method (19). Likewise, confounding variables were selected by bivariate linear models fitted by ordinary least square regressions and included in multivariate models if any adjusted-*P*-value <0.2 to eliminate sample bias. Box–Cox Power family of transformations (20) was used whenever necessary to normalize laboratory variables. Before modeling the CD4^+^ T cell counts and the CD4/CD8 ratio kinetics of the HIC individuals, the variables were log- (base 10) and square-root transformed, respectively, and then nested linear mixed-effect models (21) were fit by maximum likelihood and had their deviance compared by *F*-test with Kenward–Roger approximation. Contrasts were obtained from the fitted model to compare both CD4^+^ T cell counts and the CD4/CD8 ratio kinetics means among HIC groups of individuals. Degrees of freedom for adjusted effects were approximated by the Satterthwaite method (22). Again, *P*-values were corrected by the Tukey HSD method (18). All statistical analysis was performed in software R v. 3.4.3.

## Results

### Characteristics of the Study Groups

Table 1 summarizes the characteristics of the five study groups at a time point selected for this study (highlighted in Figure S1 in Supplementary Material). We found no dependence among the observed frequencies of any of the nominal variables evaluated (age, gender, level of education, exposure category, and time since HIV diagnosis) and the study groups, with the exception of skin color. The median follow-up time of the HIC groups was 9.02 years (IQR = 6.46). Despite no global difference among the groups, the PEC group had the highest median CD4^+^ T cell counts at the inflammation/immune activation assay date (1,243.5; IQR = 472.8). Some patients started cART due to the recommendation of the “Departamento de Vigilância, Prevenção, e Controle das IST, do HIV/Aids e das Hepatites Virais” from the Ministry of Health in Brazil, which offer cART to all PLWH.

**Table 1:** Individuals’ characteristics.

	HIV-neg (*n* = 18)	Combined antiretroviral therapy (*n* = 18)	Persistent elite controller (*n* = 8)	Ebbing elite controller (*n* = 7)	Viremic controller (*n* = 13)	*P*-value
Age (years)	37.07 (IQR = 17.76)	44.22 (IQR = 9.81)	40.82 (IQR = 8.49)	45.6 (IQR = 18.63)	41.39 (IQR = 9.25)	0.4773
Gender; *n* (%)						0.2503
Female	9 (14.1)	7 (10.9)	5 (7.8)	6 (9.4)	5 (7.8)	
Male	9 (14.1)	11 (17.2)	3 (4.7)	1 (1.6)	8 (12.5)	
Skin color; *n* (%)						0.0259
Black	2 (3.1)	1 (1.6)	0 (0)	1 (1.6)	6 (9.4)	
Brown	3 (4.7)	9 (14.1)	6 (9.4)	2 (3.1)	3 (4.7)	
White	11 (17.2)	8 (12.5)	2 (3.1)	4 (6.2)	4 (6.2)	
Exposure category; *n* (%)	NA					0.2135
Het/Other	NA	15 (23.4)	6 (9.4)	7 (10.9)	8 (12.5)	
MSM	NA	3 (4.7)	2 (3.1)	0 (0)	5 (7.8)	
Number of blips [means (IQR)]	NA	0 (IQR = 0)	0 (IQR = 0)	3 (IQR = 1.5)	12 (IQR = 8)	<0.0001
CD4^+^ T-cell count (cells/μL)	831 (IQR = 372.25)	853 (IQR = 221)	1,243.5 (IQR = 472.75)	1,027 (IQR = 505)	820 (IQR = 711)	0.1216
CD4/CD8 ratio	1.69 (IQR = 0.46)	0.95 (IQR = 0.69)	1.44 (IQR = 0.92)	1.17 (IQR = 0.41)	1.06 (IQR = 0.31)	<0.0001
CD4%	44 (IQR = 3.5)	32.95 (IQR = 7.04)	43.5 (IQR = 9.75)	44 (IQR = 11)	37 (IQR = 6)	<0.0001
Viral load (copies/mL)	NA (IQR = NA)	49 (IQR = 0)	49 (IQR = 0)	49 (IQR = 127.5)	450 (IQR = 549)	<0.0001
Time since HIV diagnosis (days)	NA	3,988 (IQR = 1,968.75)	1,694 (IQR = 4,348.25)	3,615 (IQR = 2,243.5)	4,162 (IQR = 2,970)	0.3893

*Het, heterosexual; MSM, males who have sex with males; IQR, interquartile range; NA, not applicable*.

### Most ECs Have Levels of Inflammation and Immune Activation Similar to HIV-1-Uninfected Individuals

**Figure 1:**
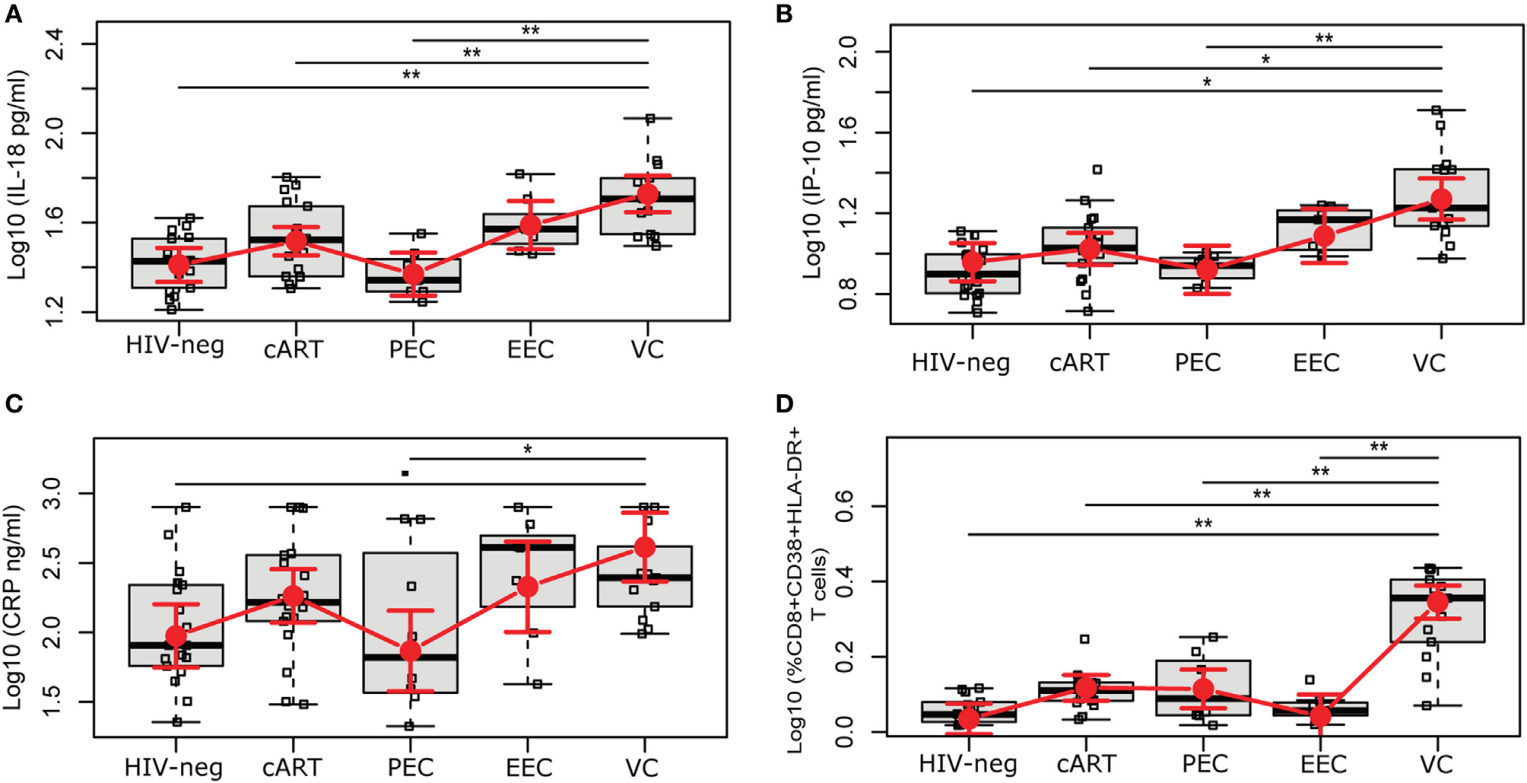
HIV-1 elite controllers have normal levels of inflammation and CD8^+^ T cell activation. HIV controllers were divided according to the level of viral controller: persistent elite controller, if 100% of VL measures were below the limit of detection; ebbing elite controller, if subjects had occasional (<30% of frequency) episodes of transient, low-level (51–400 copies/mL) viremia; and viremic controller, if most (≥70%) VL determinations were between 51 and 2,000 copies/mL. The plasmatic levels of **(A)** IL-18, **(B)** IP-10, and **(C)** CRP were measured by the ELISA test, and **(D)** the frequency of %CD8^+^CD38^+^HLA^+^DR^+^ was evaluated by flow cytometry. The time point selected for this study is highlighted in Figure S1 in Supplementary Material. The results are expressed as Log10. Boxplots represent the IQR and sample median (central solid black line). Red dots and vertical bars represent linear model estimated adjusted means and 95% confidence intervals (CI 95%). Comparisons of means among groups were performed by contrasts/differences obtained after both bi- and multivariate-linear models fitted by ordinary least square regressions. *P*-values were corrected by the Tukey Honest Significant Difference *post hoc* method, and a Type I error adjustment was conducted for multiple comparisons following the Holm-Bonferroni method. *P < 0.05, **P < 0.01.

Chronic inflammation and elevated immune activation are associated with a faster progression to AIDS and CD4^+^ T cell depletion (23, 24). Here, we evaluated the concentration of six plasmatic markers of inflammation/immune activation and frequency of activated CD8^+^ T cells (Figure 1; Figure S2 in Supplementary Material). Among the HICs, the estimated mean levels of IL-18 and IP-10 increased as the level of viremia increased (PECs < EECs < VCs), although the difference between PECs and EECs was not significant (Figures 1A,B). The estimated mean levels [95% confidence intervals], after adjustment for gender, race, education, and age, of IL-18 and IP-10 in VC (524.1 pg/mL [432.4; 634.8]; 136.3 pg/mL [137.3; 225.7]) were significantly higher than that in HIV-neg (247.3 pg/mL [206.3; 296.2]; 80.7 [63.0; 102.7]), cART (318.6 pg/mL [273.7; 370.6]; 95.3 [77.7; 116.5]), and PEC (224.1 pg/mL [177.6; 282.0]; 73.1 [53.1; 99.5]) groups. Both EC groups had levels of IL-18 and IP-10 that were not different from the HIV-neg or cART groups. VCs also presented a higher mean level of CRP (4,103.8 ng/mL [2,319.2; 7,255.9 ng/mL]) than did the PEC group (725.1 ng/mL [367.1; 1,423.2 ng/mL]) (Figure 1C). The levels of IL-6, sCD14, and sCD163 were not different among the five groups of individuals (Figure S2 in Supplementary Material).

The expression of CD38 and HLA-DR on CD8^+^ T cells has been broadly used to evaluate immune activation in HIV-1 infection (3, 10, 23, 25). The VC group presented a higher estimated mean proportion, 12.2% [10.0; 14.5], of activated CD8^+^ T cells; this proportion was significantly higher than that of the other groups (Figure 1D). The PEC (3.0% [1.6; 4.7]) and EEC (1.0% [−0.4; 2.6]) groups displayed CD8^+^ T cell estimated mean activation levels that were not different from the HIV-1-uninfected (0.8% [−0.1; 1.9]) and cART (3.1% [2.1; 4.2]) groups.

### ECs Maintain a Normal CD4/CD8 Ratio and CD4%

**Figure 2:**
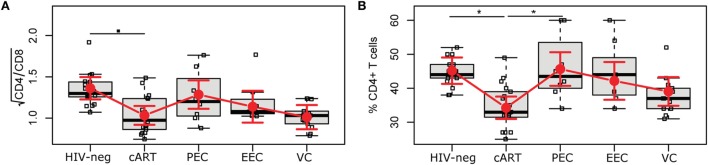
HIV controllers have a normal CD4/CD8 ratio and CD4%. The CD4/CD8 ratio **(A)** and the CD4% **(B)** were obtained using the MultiTest TruCount kit. The time point selected for this study is highlighted in Figure S1 in Supplementary Material. Boxplots represent the IQR and sample median (central solid black line). Red dots and vertical bars represent linear model estimated adjusted means and 95% confidence intervals (CI 95%). The CD4/CD8 ratio was square-root transformed; comparisons of means among groups were performed by contrasts/differences obtained after both bi- and multivariate-linear models fitted by ordinary least square regressions. *P*-values were corrected by the Tukey Honest Significant Difference *post hoc* method, and a type I error adjustment was conducted for multiple comparisons following the Holm-Bonferroni method. ^▪^*P* < 0.1, *P < 0.05.

Studies have reported an association between CD4/CD8 ratio and T cell activation (26, 27). We observed no difference of CD4/CD8 ratio (Figure 2A) among the HICs or even with the cART and HIV-1-uninfected individuals. We also evaluated the CD4% (Figure 2B), as this marker is considered one of the best predictors of AIDS-associated events (28); a previous study (10) that divided ECs based on CD4% demonstrated different levels of immune activation among the groups. The cART group showed a lower mean estimated CD4% (34.3% [31.0; 37.5]) than did the HIV-1-uninfected (45.2% [41.3; 49.1]) and PEC (45.7% [40.7; 50.6]) groups. The VC group also presented a mean adjusted CD4% <40% (39.1% [34.9; 43.3]), although this was not significantly lower than the HIV-neg or EC groups.

### No Evidence of CD4^+^ T Cell Decline Over Time in HICs

**Figure 3:**
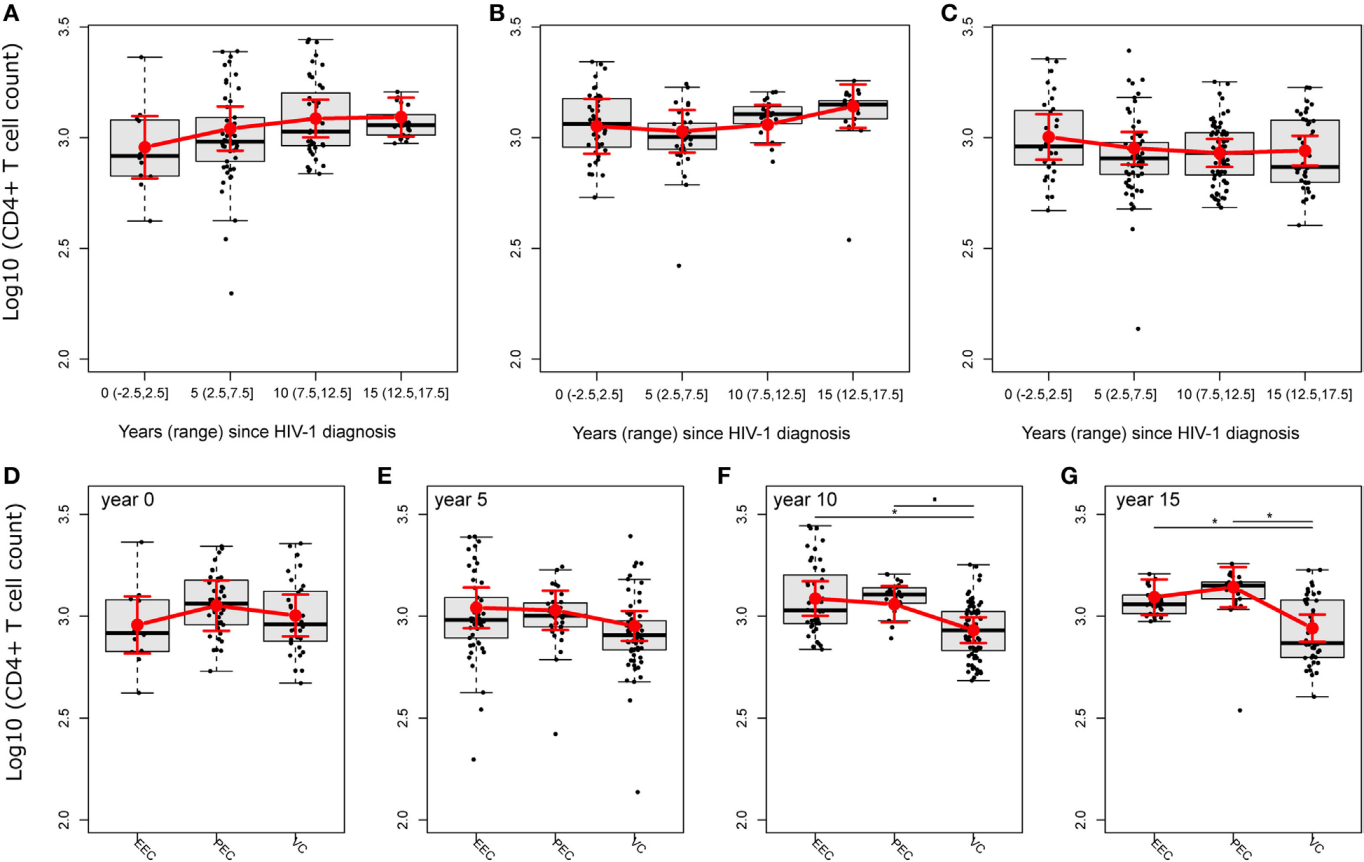
HIV controllers (HICs) have no evidence of CD4^+^ T cell decline. Fitted (sample) instant in/decrement of log_10_-transformed CD4^+^ T cell counts at 5-year intervals (ranges) after HIV-1 diagnosis for HICs, ebbing elite controller **(A)**, persistent elite controller **(B)**, and viremic controller **(C)**, and the comparison of fitted mean log_10_-transformed CD4^+^ T cell counts among HICs at year 0 **(D)**, 5 **(E)**, 10 **(F)**, and 15 **(G)** after HIV-1 diagnosis. Analysis was performed by selecting the best nested linear mixed-effect models fitted by maximum likelihood. Deviance analysis was performed among nested models by *F*-test with Kenward–Roger approximation. Slopes/contrasts were obtained from the best fitted models to compare CD4^+^ T cell counts kinetic estimated means and CI 95% (red dots and vertical bars) among HIC groups of individuals. Degrees of freedom for estimated effects were approximated by the Satterthwaite method. Boxplots represent the IQR and sample median (central solid black line). Red dots and vertical bars represent linear model estimated adjusted means and 95% confidence intervals (CI 95%). Again, *P*-values were corrected by the Tukey Honest Significant Difference. ^▪^*P* < 0.1, *P < 0.05.

The CD4^+^ T cell dynamics is an important marker of disease progression, and different studies have correlated immune activation with CD4^+^ T cell depletion (24, 29). To analyze the trajectories of CD4^+^ T cell counts and the CD4/CD8 ratio, we fitted linear mixed-effects models (Tables S1 and S2 and Figure S3 in Supplementary Material). The mean CD4^+^ T cell count (log, base 10, transformed) trajectories were distinct among the groups, but without evidence of a decline in CD4^+^ T cell counts for any group in 5 years of follow-up intervals (Figures 3A–C; Table S3 in Supplementary Material). Despite that, the fitted mean log_10_-transformed CD4^+^ T cell counts of the PEC group presented a clear, continuously rising tendency after 5 years of follow-up, while for the EEC group, this rising tendency was limited to 12.5 years, after which there was a slightly descending fitted mean. Conversely, for the VC group, the fitted mean described a much more stable pattern, with a slight descent until 12.5 years of follow-up that was reversed in the other half period. The square root of CD4/CD8 ratios, $\sqrt{\text{CD}4/\text{CD}8}$ (Table S4 in Supplementary Material) was somewhat different from the fitted mean log_10_-transformed CD4^+^ T cell count dynamics (Figure S3A in Supplementary Material). Although there is also a clear, continuously rising tendency after 5 years of follow-up for the PEC group, for the fitted mean square root of CD4/CD8 ratios (Figure S3B in Supplementary Material), we observed a more linear tendency, i.e., straightness. For the EEC group, the rising tendency for the fitted mean square root of the CD4/CD8 ratio was shorter than that for the log_10_-transformed CD4^+^ T cell counts, stopping at 5 years of follow-up, and the slight descent observed for the log_10_-transformed CD4^+^ T cell counts was much more evident and continuous for at least the remaining 3/4 of follow-up time. The more distinct pattern among HIC groups was clearly the one for the VC group (Figure S3C in Supplementary Material). We saw a clear continuously descending tendency for the fitted mean square root of the CD4/CD8 ratio that initiated right at the beginning of the observation and continued for at least 20 years. To show some of these differences along the follow-up time for both the fitted mean log_10_-transformed mean CD4^+^ T cell counts and square root of the CD4/CD8 ratio, and given the small sample size, we decided to test either if there were instantaneous in/decrements (slopes) within groups in 5-year intervals (Tables S3 and S4 in Supplementary Material), or if there were within-group differences between the beginning and 15 years of follow-up time (Tables S5 and S6 in Supplementary Material). In the former analysis, we found a significant increment of log_10_-transformed mean CD4^+^ T cell counts for the EEC group at 5 years of follow-up (log_10_Count = 0.05 [0.012; 0.088]; Adj-*P*-Val = 0.047), which is equivalent of a mean increment of 12.24% [2.89%; 22.44%] CD4^+^ T cells. No significant in/decrement was observed for the PEC or VC fitted log_10_-transformed CD4^+^ T cell count mean at 0, 5, 10, or 15 years of follow-up. Also, no significant in decrement was observed for any HIC group for the fitted mean square root of the CD4/CD8 ratio. In the second analysis, the EEC group had a tendency of increase of the log_10_-transformed CD4^+^ T cell count (Figures 3A–C; Table S5 in Supplementary Material) after 15 years of follow-up (log_10_FC = 0.136 [−0.005; 0.277]; Adj-*P*-Val = 0.0584). No difference was observed for the PEC or VC fitted log_10_-transformed CD4^+^ T cell count mean after 15 years of follow-up. The PEC group showed a significant fitted mean square root of CD4/CD8 ratio increase after 15 years of follow-up (Figures S4A–C and Table S6 in Supplementary Material) (Diff. = 0.224 [0.026; 0.423]; Adj-*P*-value = 0.0289). No difference was observed for the EEC or VC fitted mean square root of the CD4/CD8 ratio after 15 years of follow-up (Figures S4A–C and Table S6 in Supplementary Material). We then decided to compare both the fitted mean log_10_-transformed mean CD4^+^ T cell counts and fitted mean square root of the CD4/CD8 ratio among groups (Figures 3D–G). In these analysis, we observed differences between PECs and VCs after 10 years of follow-up (log_10_FC = 0.128 [0.014; 0.241]; Adj-*P*-Val = 0.0727) and EECs and VCs (log_10_FC = 0.155 [0.044; 0.266]; Adj-*P*-Val = 0.021) (Figure 3F; Table S7 in Supplementary Material). Differences between both PECs and VCs (log_10_FC = 0.201 [0.073; 0.329]; Adj-*P*-Val = 0.01) and EECs and VCs (log_10_FC = 0.152 [0.033; 0.271]; Adj-*P*-Val = 0.0378) were also observed after 15 years of follow-up (Figure 3G; Table S7 in Supplementary Material). Once VCs had no evidence of CD4^+^ T cell count decrease, these results were possibly due to an increase of CD4^+^ T cell counts in EECs. Using resembling analyses (Figures S4D–G and Table S8 in Supplementary Material), we observed both a difference after 10 years of follow-up between the fitted mean square root of CD4/CD8 ratios of PECs and VCs (Figure S4F and Table S8 in Supplementary Material) (Diff. = 0.249 [0.054; 0.444]; Adj-*P*-Val = 0.036) and a borderline difference between EECs and VCs (Figure S4F and Table S8 in Supplementary Material) (Diff. = 0.217 [0.024; 0.41]; Adj-*P*-Val = 0.072). The difference between PECs and VCs was also observed after 15 years of follow-up (Figure S4G in Supplementary Material) (Diff. = 0.373 [0.134; 0.611]; Adj-*P*-Val = 0.009). No differences of the fitted mean square root of CD4/CD8 ratios among groups were observed at the beginning or after 5 years of follow-up.

### IL-18 and IP-10 Correlate With Cumulative Viral Load (cVL), CD4/CD8 Ratio, and CD4%

**Figure 4:**
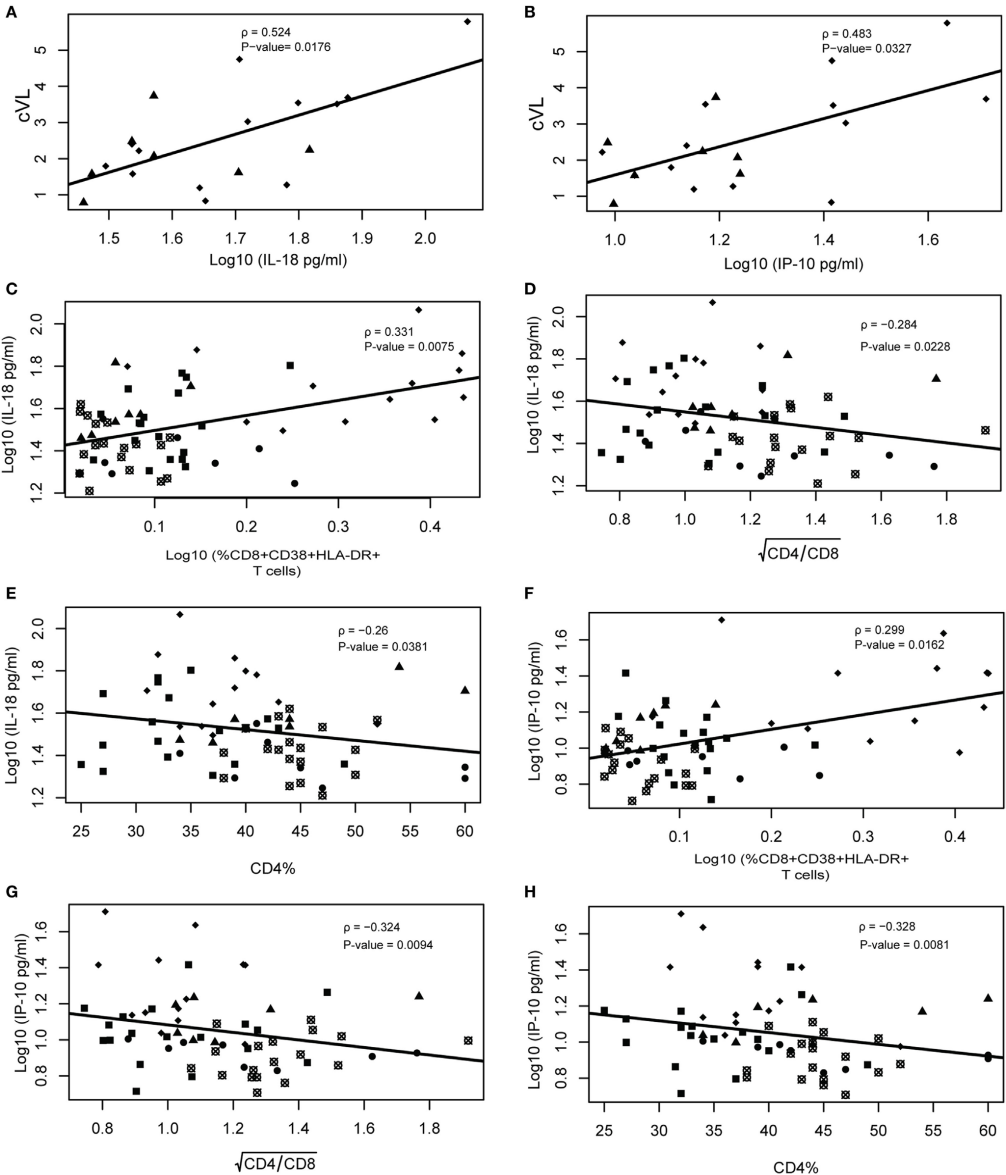
IL-18 and IP-10 correlate with cumulative viral load, CD4/CD8 ratio, and CD4%. Viral load correlations were performed only using ebbing elite controller (EEC) and viremic controller (VC) groups. Spearman rank correlation of IL-18 vs cumulative viral load (cVL) **(A)** and IP-10 vs cVL/year **(B)**. Correlation between IL-18 vs %CD8^+^CD38^+^HLA^+^DR^+^ **(C)**, $\sqrt{\text{CD}4/\text{CD}8}$ **(D)**, and %CD4 **(E)**; and between IP-10 vs %CD8^+^CD38^+^HLA^+^DR^+^ **(F)**, $\sqrt{\text{CD}4/\text{CD}8}$ **(G)**, and %CD4 **(H)**. Spearman’s rank correlations coefficient analysis was performed among these variables with type I error adjustment for multiple comparisons following the Holm–Bonferroni method. Crossed open black circles, HIV-neg; solid black squares, combined antiretroviral therapy; solid circles, persistent elite controller; solid black triangles, EEC; and solid black lozenges, VC.

We analyzed the correlation between cVL, CD4%, CD4/CD8 ratio, and markers of inflammation or immune activation. When considering only EECs and VCs, both IP-10 (ρ = 0.4830; *P*-value = 0.0327) and IL-18 (ρ = 0.5140; *P*-value = 0.0176) presented a moderate positive linear relationship with cVL (Figures 4A,B), and no correlation was observed between CD8^+^ T cell activation and cVL. When considering all HICs, the HIV-neg group and cART groups, IL-18 and IP-10 correlated positively with CD8^+^ T cell activation (ρ = 0.3310; *P*-value = 0.0075 and ρ = 0.2990; *P*-value = 0.0162, respectively) and negatively with CD4/CD8 (ρ = −0.2840; *P*-value = 0.00228 and ρ = −0.3240; *P*-value = 0.0094, respectively) (Figures 4C,F) and CD4% (ρ = −0.2600; *P*-value = 0.0381 and ρ = −0.3280; *P*-value = 0.0081, respectively) (Figures 4D,E,G,H). CD8^+^ T cell activation presented a weak correlation with CD4/CD8 ratio and CD4% (ρ = −0.2950; *P*-value = 0.0179 and ρ = −0.2490; *P*-value = 0.0474, respectively) (Figures S5A,B in Supplementary Material). We also observed a correlation between IP-10 and IL-18 with CD8^+^ T cell activation (Figures S5C,D in Supplementary Material).

### Some ECs Displayed an Immune Profile Indistinguishable From HIV-1 Uninfected Individuals

**Figure 5:**
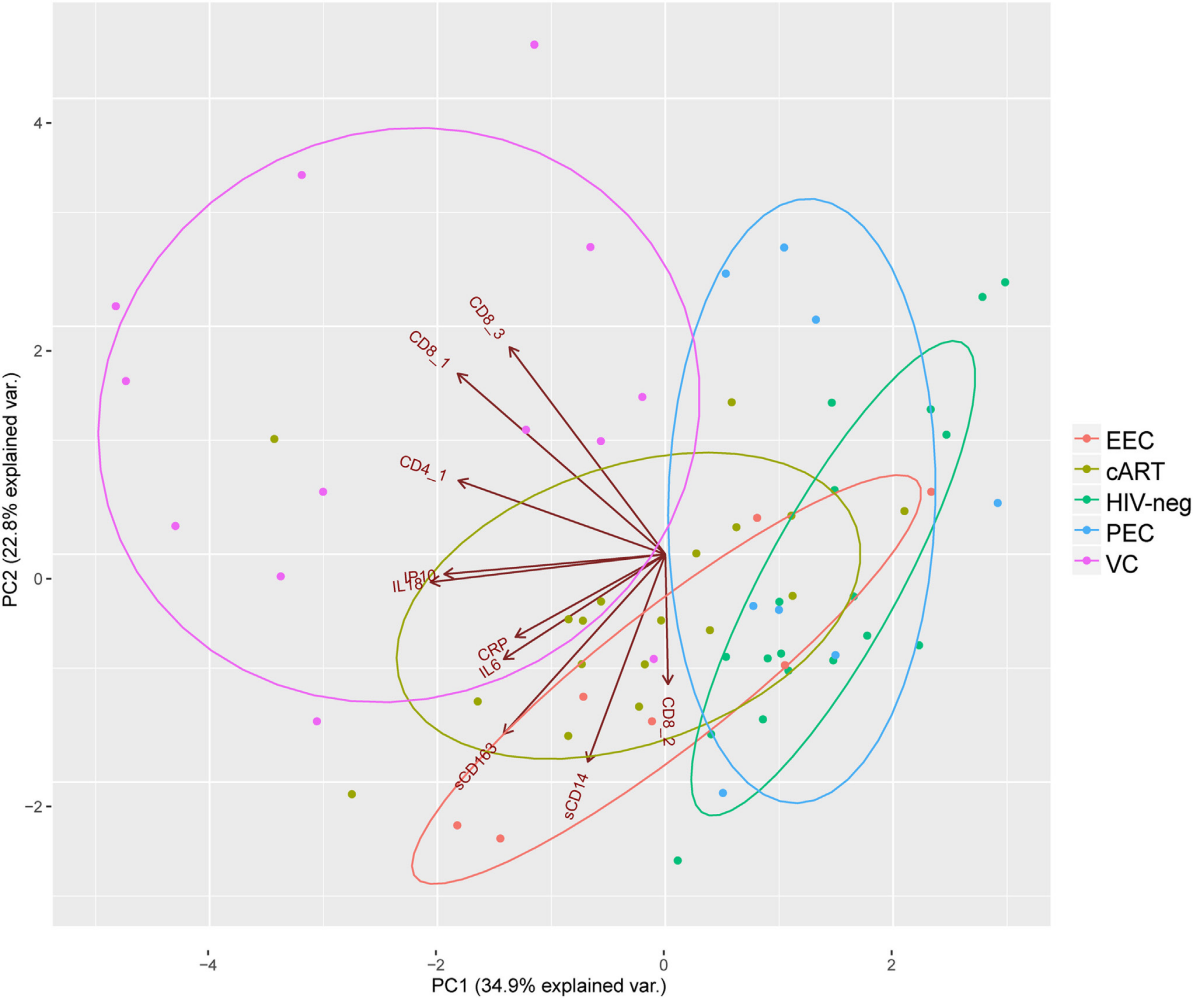
Principal component analysis highlights elite controllers with an immune profile similar to HIV-1 negative individuals: CD8_1, CD8^+^CD38^+^HLA-DR^+^; CD8_2, CD8^+^HLA-DR^+^; CD8_3, CD8^+^CD38^+^; CD4_1, CD4^+^CD38^+^HLA-DR^+^. Brown directed vectors represent the contribution of each variable for the formation of the two-principal components. Colored ellipses represent the 68% of the normal concentration for each group.

Elite controllers are considered models of HIV control, and their immunological profiles could give insights to identify biomarkers of an HIV functional cure (30). Here, we performed PCA combining the different immunological characteristics analyzed among the five groups included in this study (Figure 5). The VC individuals are the more disperse among the groups and are totally separated from HIV-1-uninfected individuals and EECs, and they have almost no superposition with PECs. The cART individuals have a very limited superposition with HIV-1 negative individuals, reinforcing the idea that despite prolonged viral suppression, the most treated individuals do not achieve a normal immunological profile. Half of the PECs and two EECs grouped with HIV-negative individuals, highlighting the preserved immunological profile of these individuals. IL-18 and IP-10 concentration and CD8^+^ T cell activation have the highest contribution to the formation of the first principal component, which is alone responsible for explaining 39.4% of the data covariance. The combined analysis of these inflammatory/activation markers suggests the existence of two subgroups of ECs. One subgroup (EC^low^) displayed inflammatory and activation markers within the normal range, while the other subgroup (EC^high^) displayed inflammatory and/or activation markers that were above the normal range (Table S9 in Supplementary Material).

## Discussion

In this study, we evaluated inflammation, CD8^+^ T cell activation, CD4%, and CD4^+^/CD8^+^ T cell dynamics in HICs with long-term (>5 years) viral control at different levels. We observed that long-term ECs with undetectable viremia (≤50–80 copies/mL) presented an inflammation and activation profile similar to that observed among HIV-1 negative individuals, in agreement with previous studies using comparable stringent criteria for EC definition (10–12). In sharp contrast, we found that a persistent low-level viremia (80–2,000 copies/mL) is sufficient to drive elevated chronic levels of immune activation and inflammation in long-term VCs.

Plasmatic levels of IP-10 and IL-18 correlated with cVL control and CD8^+^ T cell activation in our HICs cohort. There is increasing evidence of the positive relationship between IP-10 and HIV-1 VL (31–33), and previous studies have demonstrated a higher level of IP-10 in some ECs than in HIV-negative individuals (25, 34). Thus, the normal level of IP-10 in our ECs could reflect an extremely low level of residual viral replication, consistent with the very low frequency of HIV-1 Gag-specific responses in the IFN-γ ELISPOT assay previously described for some of these ECs (11). HIV-1-infected individuals present higher levels of IL-18 than do HIV-uninfected individuals (35–37). To the best of our knowledge, only two previous studies analyzed the IL-18 levels in HICs (38, 39) but did not compare EC with HIV-1 uninfected individuals.

Our analyses also support that inflammation and T cell activation reflects the past cumulative viral replication better than VL at a single point as we did not observe a direct linking between the presence of VL blips and higher levels of inflammation and/or T cell activation in EC. For instance, individual EEC36 was analyzed during a blip (1,086 copies/mL), but displayed a low cVL (3.17 copies/mL/year) and one of the lowest levels of inflammation (IP-10 = 89.39 pg/mL and IL-18 = 278.44 pg/mL) and T cell activation (0.47%). On the other hand, individual EEC18 was analyzed at a point with undetectable VL, but displayed a relative high cVL (8.47 copies/mL/year) and a high levels of inflammation (IP-10 = 161.73 pg/mL and IL-18 = 362.62 pg/mL) and T cell activation (2.15%) among EC.

Although the mean levels of inflammatory and activation markers in our EC cohort were similar to those observed in the HIV-uninfected group, the combined analysis of the inflammatory markers IP-10 and IL-18 and of activation marker CD8^+^CD38^+^HLA-DR^+^ T cells suggest the existence of two subgroups of ECs. The subgroup EC^low^ displayed inflammatory and activation markers indistinguishable from HIV-uninfected subjects and probably represent the best model of natural functional cure. The subgroup EC^high^, by contrast, displayed inflammatory and/or activation markers that were above the normal range. Interestingly, the EC^low^ and EC^high^ subgroups did not match the PEC and EEC subgroups, suggesting that some EC are able to maintain normal levels of inflammatory and activation markers despite occasional blips, while some ECs display high activation despite persistent undetectable viremia. Further studies using longitudinal analyses and a higher number of individuals will certainly contribute to a better definition of these groups.

The pathogenic effect of the high levels of CD8^+^CD38^+^HLA-DR^+^ T cells observed in three PECs, however, should be interpreted with caution. In sharp contrast to the activation profile typically observed in HIV-1 infection, the three PEC subjects presenting the highest expression of HLA-DR in CD8^+^ T cells also showed very low expression of CD38. Notably, HIV-1 specific CD8^+^ T cells expressing only HLA-DR present a better functional profile (higher frequency of IFN-γ^+^IL-2^+^-producing cells, higher proliferative and cytotoxic capacities) than those expressing both HLA-DR and CD38 (16, 40, 41). Although we only evaluated CD38 and HLA-DR expression in the bulk CD8^+^ T cells, these observations suggest that the high levels of CD8^+^HLA-DR^+^ T cells detected in some PECs may reflect a more functional anti-HIV CD8^+^ T cell condition, instead of a harmful non-specific immune activation. However, more analyses are necessary to test this hypothesis, as compare CD107a, IFN-γ, and IL-2 expression, with a HIV-1 specific stimulus, between CD8^+^HLA-DR^+^ and CD8^+^HLA-DR^-^ T cells in these patients.

Some studies have reported CD4^+^ T cell depletion in some HICs (3–5, 42, 43), although the frequency of this phenomenon greatly varies among the different cohorts evaluated, mainly due to the criteria of HIC definition and the number of subjects evaluated. Leon et al. (42), using the criteria of VL <50 copies/mL (ECs) and <2,000 copies/mL (VCs) for at least 1 year, identified 475 HICs and showed a significant CD4^+^ T cell decline in 48% of them. By contrast, a study including 217 HICs from the ANRS C021 CODEX (43) with VL <400 copies/mL and at least 5 years of HIV-1 diagnosis found that only 5% experienced immunological progression. We detected no evidence of CD4^+^ T cell depletion in any of the long-term HIC groups evaluated in our study. This suggests that CD4^+^ T cell depletion may be a rare phenomenon in HICs maintaining viral suppression for more than 5 years, even for VCs and EC^high^ subgroups.

Beyond the CD4^+^ T cell decrease, one of the characteristics of HIV-1 infection is CD8^+^ T cell increase, resulting in a lower CD4/CD8 ratio and CD4% over time (44). The CD4/CD8 ratio predicts the time to AIDS in HIV-1-infected untreated individuals (26), and a low CD4/CD8 ratio (0.51–0.80) is associated with an increased risk of losing virological control in HIC individuals (45). The CD4% is also one of the best predictors of AIDS-related events, even after therapy start (34), and it was highlighted that ECs with CD4% >40% had normal levels of T cell activation and reduced expression of exhaustion markers compared with ECs presenting CD4% <40% (10). All ECs included in our study displayed a median CD4/CD8 ratio >1 and a median CD4% >40%, except for one PEC individual. Half of the VC subjects, by contrast, displayed a CD4/CD8 ratio <1 (47%) and CD4% <40% (86%). Thus, although we detected no evidence of CD4^+^ T cell depletion in the HIC groups evaluated in our study, persistent low-level viremia in VC appears to be associated with CD8^+^ T cell increase and consequently lower CD4/CD8 ratio and CD4% over time.

Our study has some limitations. First, the small number of HICs evaluated may have imposed a limitation on our capacity to detect significant differences between groups, although that limitation was compensated by the more stringent criteria of HIC definition and long-term follow-up. Second, an ultrasensitive VL assay could permit a better characterization of the accumulative residual viremia in our ECs and its relationship with markers of inflammation and immune activation. Finally, we classified the group as EC^low^ and EC^high^ based in only one point, a longitudinal study could help to minimize transient fluctuations in the levels of inflammation and T cell activation within each individual and to identify better plasmatic biomarkers of functional cure in ECs.

Taken together, our results confirm that the use of more stringent criteria for EC classification, combined plasmatic levels of IL-18 and IP-10, and a frequency of CD8^+^CD38^+^HLA-DR^+^ T cells could help to identify individuals with true preserved immune integrity, which is essential in the search for correlates of a functional cure in HIV-1 infection. The lack of CD4^+^ T cell depletion in all long-term ECs analyzed here raises the question about the benefits of cART for this population, especially for those EC subjects with normal immune activation and inflammation profiles. This study reinforced the notion that initiation of cART in ECs should be evaluated individually and supports the importance of EC follow-up with elevated plasmatic levels of IL-18, IP-10, and activated CD8^+^ T cells to identify those ECs at most risk for disease progression.

## Ethics Statement

All participants provided written informed consent, and the ethical committee of Instituto Nacional de Infectologia Evandro Chagas (INI-Fiocruz) approved the study (CAAE 1717.0.000.009-07).

## Author Contributions

FC, GB, and MM conceptualized and designed the study. FC, GB, MM, and MG contributed to the experimental design and provided intellectual input; FC, DC, and SA performed sample processing, clinical data, and intellectual input. FC and HP performed the experiments. ST performed the genetic analyses of HICs. BH, BG, and VV included patients and provided clinical data. FC, HP, GB, and MR-A analyzed data and wrote the manuscript.

## Conflict of Interest Statement

The authors declare that the research was conducted in the absence of any commercial or financial relationships that could be construed as a potential conflict of interest.
