# Supplementary material for: Plasmatic Levels of IL-18, IP-10, and Activated CD8+ T Cells Are Potential Biomarkers to Identify HIV-1 Elite Controllers With a True Functional Cure Profile
Source: Front Immunol. 2018 Jul 11;9:1576. doi: 10.3389/fimmu.2018.01576 (PMC6050358; doi:10.3389/fimmu.2018.01576)
Supplement: Supplementary file 1 [file data_sheet_1.docx]

**
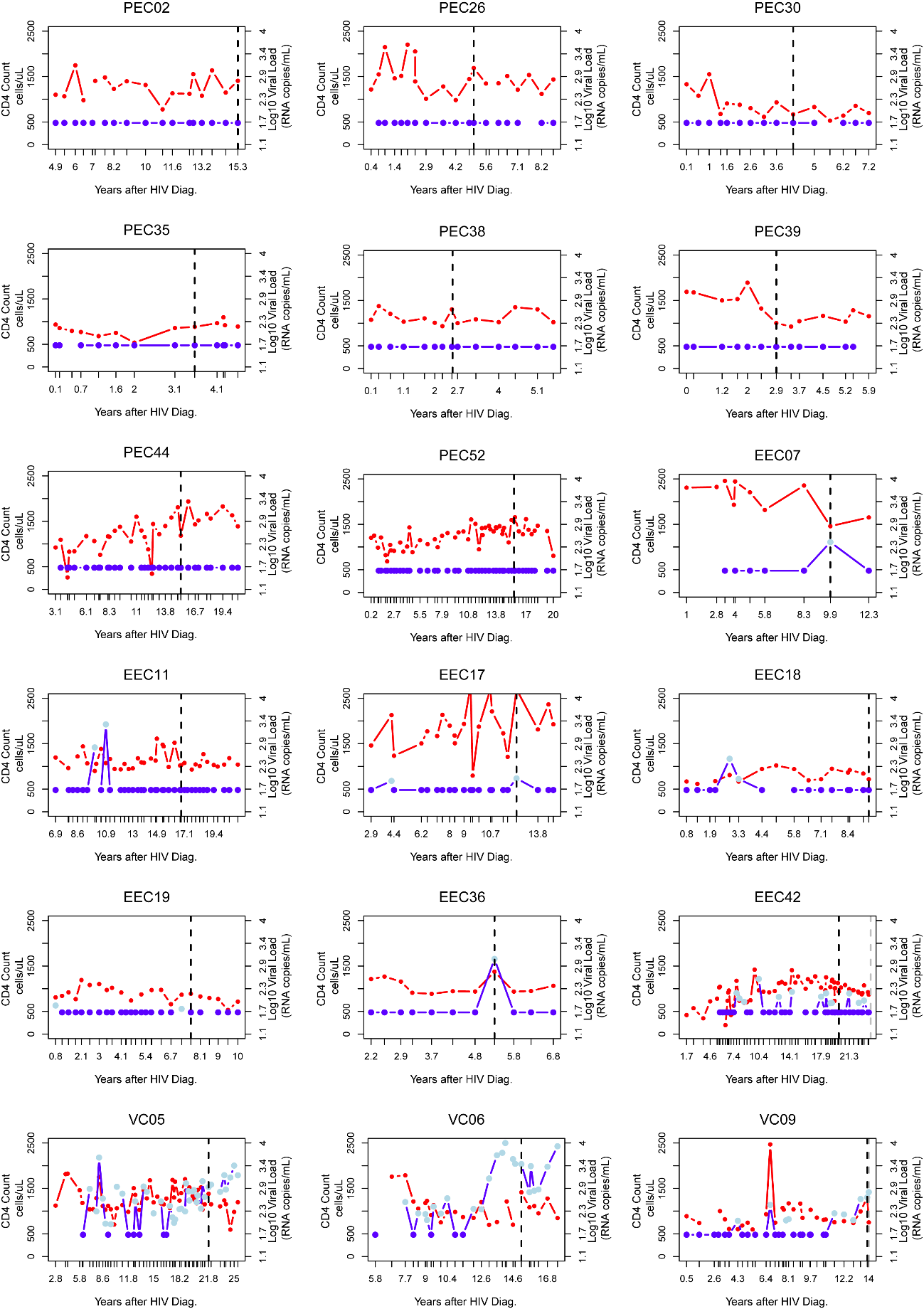
**

**
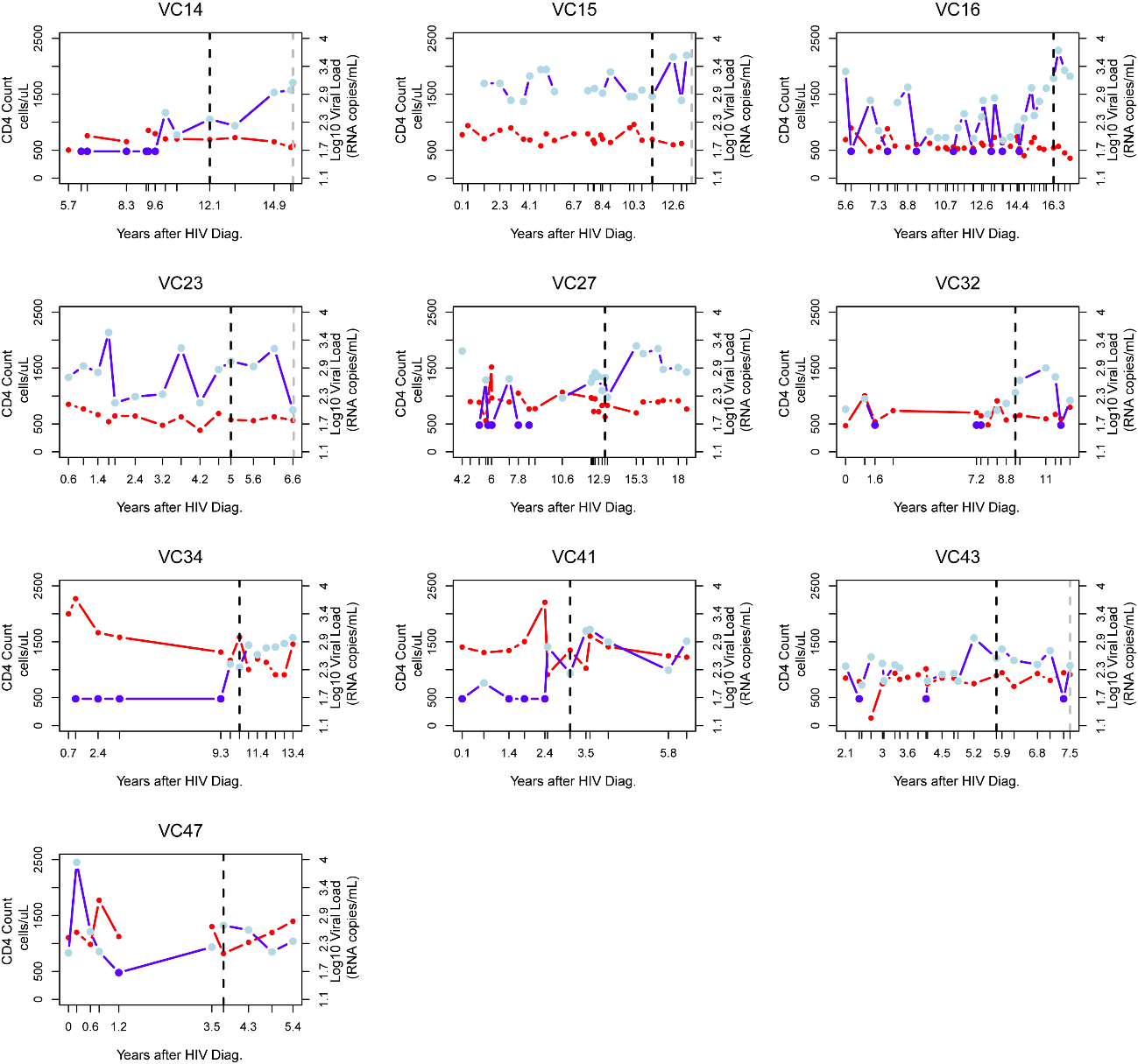
**

**Figure S1 | Kinetic of CD4^+^ T cell counts and HIV-1 viral load.** HICs were divided according to the level of viral controller: PEC, if 100% of VL measures were below the limit of detection; EEC, if subjects had occasional (< 30% of frequency) episodes of transient low-level (51–400 copies/mL) viremia; and VC, if most (≥ 70%) VL determinations were between 50 and 2,000 copies/mL. CD4^+^ T cell counts are shown on the left Y axis in red. HIV-1 viral loads are presented on the right Y axis in blue, light blue circles represent VL points above the limit of detection. The black dashed lines indicate the point evaluated and the gray dashed lines indicated the time of cART start.


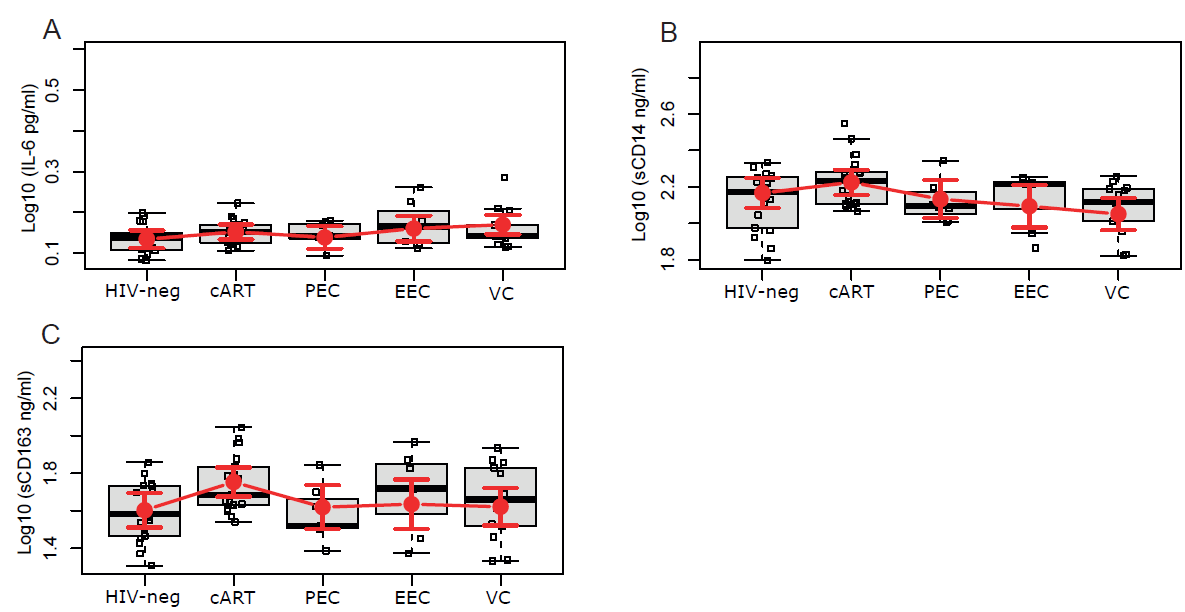


**Figure S2 | IL-6, sCD14, and sCD163 were similar among the groups evaluated.** The plasmatic levels of **(A)** IL-6 **(B)** sCD14 and **(C)** sCD163 were measured by ELISA assay. The time point selected for this study is highlighted in Figure S1 in Supplementary Material. The results are expressed as Log10. Boxplots represent the IQR and sample median (central solid black line). Red dots and vertical bars represent linear model estimated adjusted means and 95% confidence intervals (CI 95%). Comparisons of means among groups were performed by contrasts/differences obtained after both bi- and multivariate linear models fitted by ordinary least square regressions. P-values were corrected by the Tukey Honest Significant Difference post hoc method, and a Type I error adjustment was conducted for multiple comparisons following the Holm-Bonferroni method.


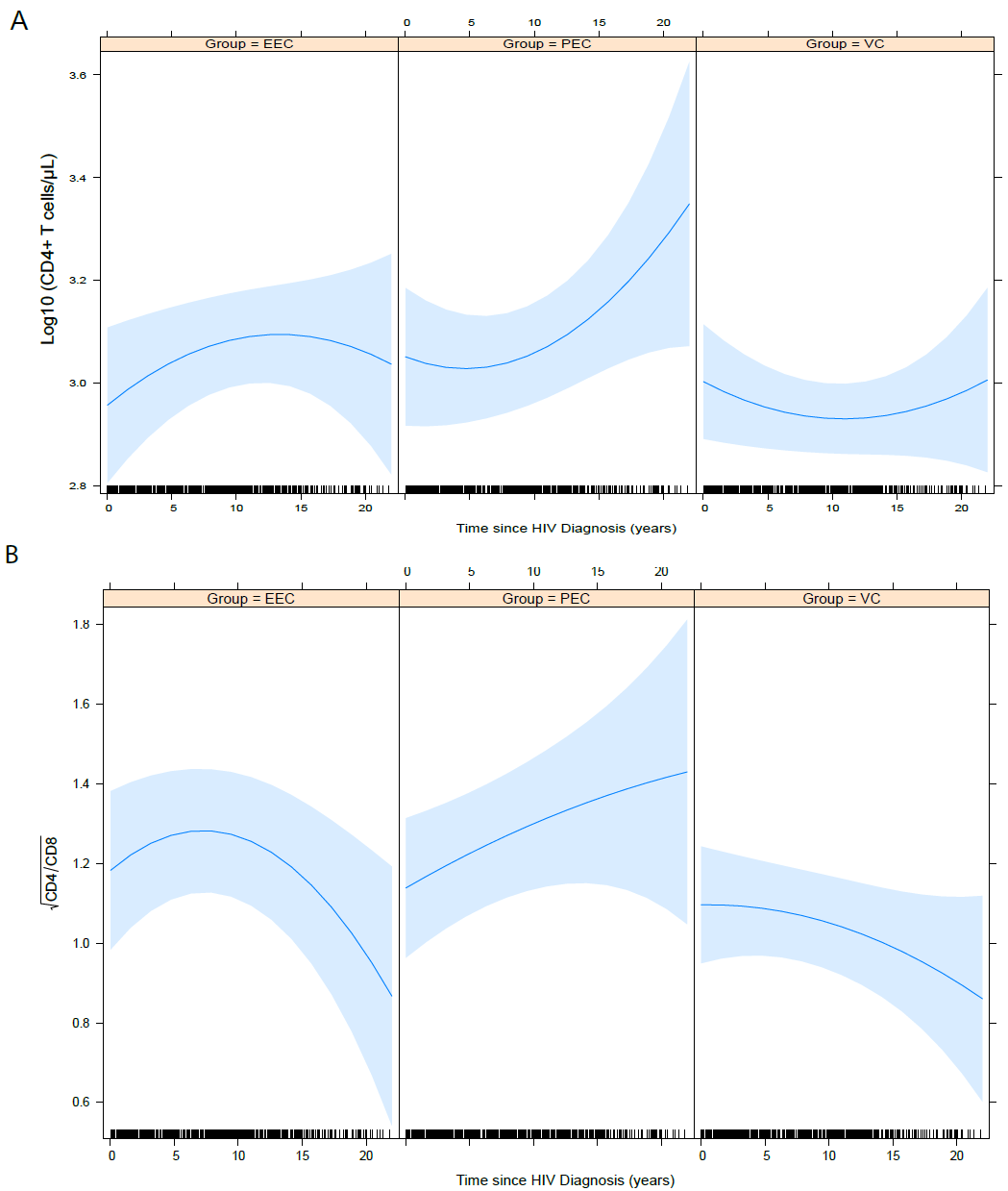


**Figure S3 | Kinetics of CD4^+^ T cell counts and CD4/CD8 ratio.** CD4^+^ T cell counts were Log10 transformed and CD4/CD8 ratio was square-root transformed. Linear mixed-effects models were fitted to analyze the dynamics of CD4^+^ T cell counts (A) and CD4/CD8 ratio (B). Blue solid lines indicate the fitted mean (point-estimation), while light blue areas represent the 95% confidence interval of the fitted mean.


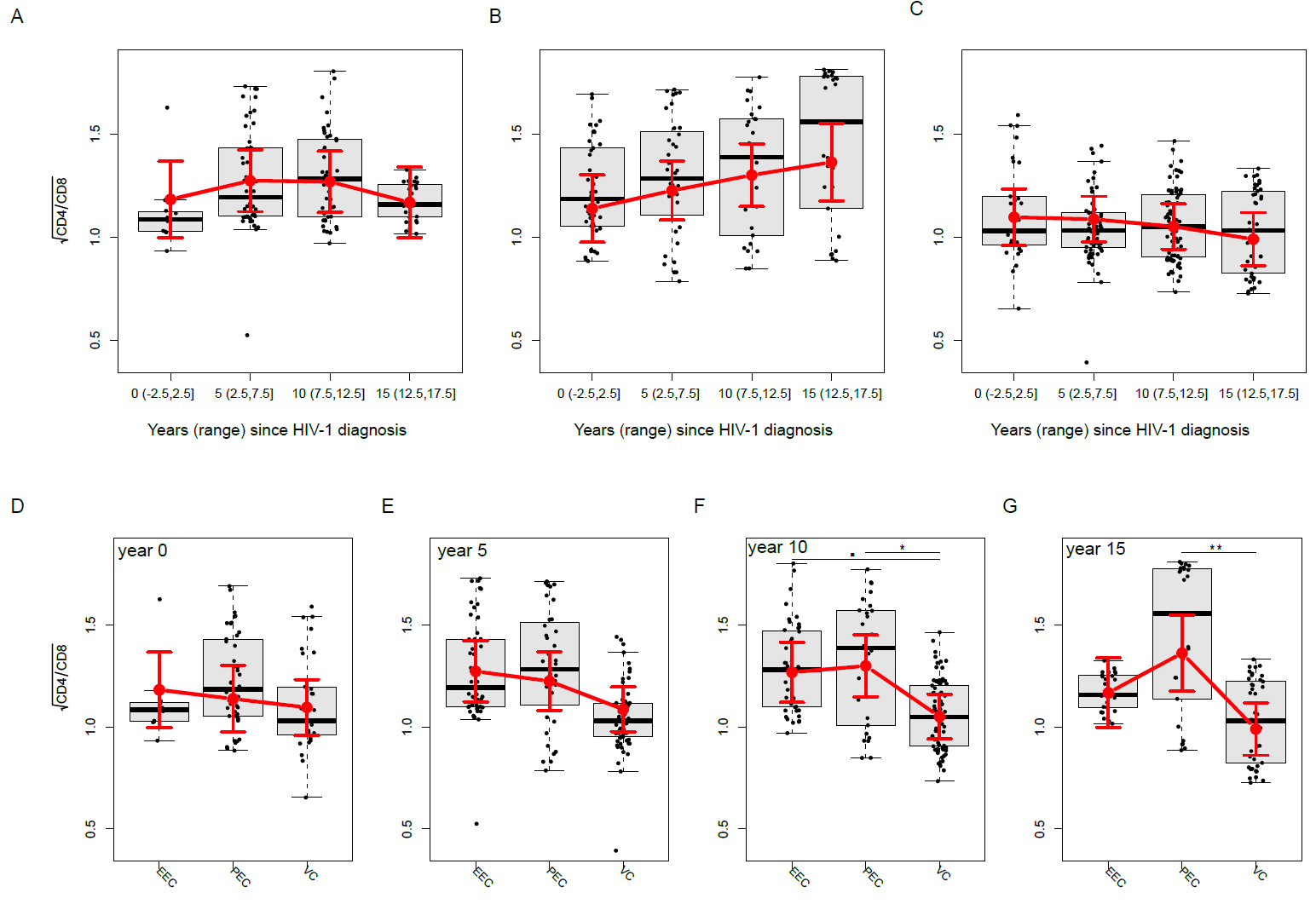


**Figure S4 | Kinetics of CD4/CD8 ratio.** CD4/CD8 ratio was square-root transformed. The kinetics of CD4/CD8 ratio in EEC (A), PEC (B) and VC (C), and the comparison of instant slope among HICs at year 0 (D), 5 (E), 10 (F) and 15 (G) years were performed by selecting the best nested linear mixed-effects model fitted by maximum likelihood. Deviance analysis was performed among nested models by F-test with Kenward-Roger approximation. Contrasts were obtained from the best fitted models to compare either CD4+ T cell counts or the CD4/CD8 ratio kinetic estimated means and CI 95% (red dots and vertical bars) among HIC groups of individuals. Degrees of freedom for estimated effects were approximated by the Satterthwaite method. Sample medians and IQR are represented by central black solid bars and boxplots. Again, P-values were corrected by the Tukey HSD. ▪ p < 0.1, *p < 0.05.


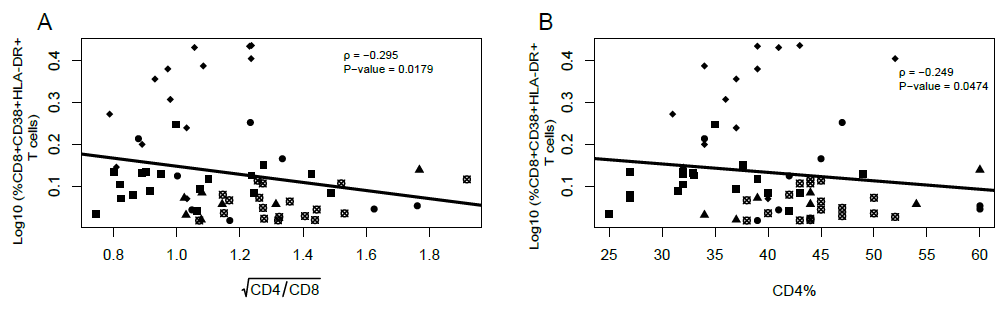


**Figure S5 | Correlation between CD8^+^ T cell activation and square root of CD4/CD8 ratio and CD4%.** HICs were divided according to the level of viral controller: PEC, if 100% of VL measures were below the limit of detection; EEC, if subjects had occasional (< 30% of frequency) episodes of transient low-level (51–400 copies/mL) viremia; and VC, if most (≥ 70%) VL determinations were between 50 and 2,000 copies/mL. We tested the correlation between the frequency of CD8^+^CD38^+^HLA-DR^+^ T cell with CD4/CD8 ratio, square-root transformed (A) and CD4% (B). Spearman's Rank Correlations Coefficient analysis was performed among these variables with Type I error adjustment for multiple comparisons following the Holm-Bonferroni method. Crossed open black circles, HIV-neg; solid black squares, cART; solid circles, PEC; solid black triangles, EEC; solid black lozenges, VC.

**Table S1:** Fixed-effect parameters of the CD4+ T cell counts (log_10_-transformed) estimated by linear mixed-effects model.

| Term | Parameters | (log_10_)^-1^ Parameters | SE | (log_10_)^-1^ SE | p.value |
| --- | --- | --- | --- | --- | --- |
| (Intercept) | 3.053254064 | 1130.457043 | 0.044754721 | 1.108548556 | <0.0001 |
| poly(Year, 2)1 | 0.756359256 | 5.70636117 | 0.505139997 | 3.199926452 | 0.136910789 |
| poly(Year, 2)2 | -0.517992878 | -0.303394094 | 0.286023525 | 1.93207297 | 0.072617752 |
| GroupPEC | 0.018861664 | 1.044387496 | 0.062547895 | 1.154909344 | 0.763508204 |
| GroupVC | -0.10149842 | -0.791592335 | 0.055668707 | 1.1367598 | 0.070731128 |
| poly(Year, 2)1:GroupPEC | 0.402149766 | 2.52435114 | 0.732755631 | 5.404501366 | 0.584140701 |
| poly(Year, 2)2:GroupPEC | 1.231345117 | 17.03511685 | 0.495889848 | 3.132491118 | 0.014393233 |
| poly(Year, 2)1:GroupVC | -0.996033471 | -0.100917511 | 0.633393874 | 4.299261623 | 0.118436218 |
| poly(Year, 2)2:GroupVC | 0.927341682 | 8.459441325 | 0.371991424 | 2.35500278 | 0.014020912 |

Standard error, SE; poly()n, polynomial of the n^th^ degree.

**Table S2:** Fixed-effect parameters of the CD4/CD8 ratio (square-root transformed) estimated by linear mixed-effects model.

| Term | Parametersfixef(mod7)fixef (mod8) | inv(log_10_)^-1^ Parameters(fixef(mod7))invsqrt (fixef(mod8)) | SEstd.errorstd.error | (invlog_10_ )^-1^ SE(std.error)invsqrt (std.error) | p.value |
| --- | --- | --- | --- | --- | --- |
| (Intercept) | 1.227130272 | 1.505848704 | 0.0752161 | 0.005657467 | <0.0001 |
| poly(Year, 2)1 | -0.913499941 | -0.834482141 | 0.6917875 | 0.47856995 | 0.19540266 |
| poly(Year, 2)2 | -1.267087299 | -1.605510224 | 0.3563688 | 0.126998754 | 0.00112307 |
| GroupPEC | 0.043500075 | 0.001892257 | 0.1057278 | 0.011178365 | 0.68330995 |
| GroupVC | -0.175866816 | -0.030929137 | 0.0935705 | 0.008755433 | 0.0686802 |
| poly(Year, 2)1:GroupPEC | 2.568642216 | 6.597922832 | 1.0481955 | 1.09871374 | 0.01950759 |
| poly(Year, 2)2:GroupPEC | 1.105962791 | 1.223153695 | 0.5300715 | 0.280975843 | 0.04443302 |
| poly(Year, 2)1:GroupVC | -0.135817322 | -0.018446345 | 0.8728256 | 0.76182448 | 0.87725414 |
| poly(Year, 2)2:GroupVC | 0.922743779 | 0.851456081 | 0.4628297 | 0.214211353 | 0.05417394 |

**Table S3:** Estimated instant in-/de-creases (slopes) of CD4^+^ T (log_10_-transformed) cell counts at year 0, 5, 10 and 15.

| Slope at  Year | Group | estimate | SE | df | t.ratio | p.value |
| --- | --- | --- | --- | --- | --- | --- |
| 0 | EEC | -0.040534634 | 0.048790798 | 30.03740438 | -0.830784391 | 0.412650647 |
| 5 | EEC | 0.050153747 | 0.018090694 | 19.69954926 | 2.772350575 | **0.047449905** |
| 10 | EEC | 0.04534419 | 0.021762033 | 61.26062952 | 2.08363762 | 0.082744302 |
| 15 | EEC | -0.054963304 | 0.044063629 | 16.64095586 | -1.24736215 | 0.306045936 |
| 0 | PEC | -0.118295089 | 0.048814428 | 25.27222365 | -2.423363199 | 0.065023118 |
| 5 | PEC | -0.031335933 | 0.021996465 | 28.9931786 | -1.424589472 | 0.164951641 |
| 10 | PEC | 0.043479578 | 0.021136862 | 94.82145006 | 2.05704978 | 0.065023118 |
| 15 | PEC | 0.106151444 | 0.049940775 | 16.7065201 | 2.12554658 | 0.065023118 |
| 0 | VC | 0.04015168 | 0.037178572 | 30.81050116 | 1.079968315 | 0.38471743 |
| 5 | VC | 0.030685567 | 0.014530609 | 22.25016436 | 2.111788035 | 0.134803336 |
| 10 | VC | -0.004733057 | 0.017027557 | 65.17430527 | -0.277964519 | 0.781919506 |
| 15 t | VC | -0.06610419 | 0.033850391 | 17.10389129 | -1.952833875 | 0.134803336 |

Standard error, SE; poly()n, polynomial of the n^th^ degree.

**Table S4:** Estimated instant in-/de-creases (slopes) of CD4/CD8 ratio (square-root transformed) at year 0, 5, 10 and 15.

| Slope at  Year | Group | estimate | SE | df | t.ratio | p.value |
| --- | --- | --- | --- | --- | --- | --- |
| 0 | EEC | -0.087427761 | 0.039211423 | 42.79904038 | -2.229650276 | 0.062140607 |
| 5 | EEC | -0.003312539 | 0.012554752 | 11.36082053 | -0.263847461 | 0.796622263 |
| 10 | EEC | 0.042057611 | 0.018166302 | 107.8949343 | 2.315144348 | 0.062140607 |
| 15 | EEC | 0.04868269 | 0.031943036 | 13.28174979 | 1.524047052 | 0.201264789 |
| 0 | PEC | -0.018650379 | 0.036587912 | 29.31147562 | -0.509741566 | 0.614049352 |
| 5 | PEC | -0.041788561 | 0.017372588 | 42.96084508 | -2.40543089 | 0.082126997 |
| 10 | PEC | -0.011569091 | 0.019448398 | 166.8333992 | -0.59486086 | 0.614049352 |
| 15 | PEC | 0.072008031 | 0.033307285 | 13.27257395 | 2.161930396 | 0.098891529 |
| 0 | VC | 0.046461595 | 0.028670212 | 34.2116505 | 1.620552863 | 0.228591653 |
| 5 | VC | -0.004925243 | 0.010401741 | 12.98151094 | -0.473501799 | 0.643719027 |
| 10 | VC | -0.025693419 | 0.013725698 | 65.46936338 | -1.871920735 | 0.228591653 |
| 15 | VC | -0.015842933 | 0.024111934 | 13.21844431 | -0.657057764 | 0.643719027 |

Standard error, SE; degrees of freedom, df; T statistic; t. ratio.

**Table S5:** Estimated contrasts/differences between year 15 and year 0 of CD4^+^ T cell counts.

| contrast | Group | estimate | SE | df | t.ratio | p.value |
| --- | --- | --- | --- | --- | --- | --- |
| 0 - 15 | EEC | -0.136110451 | 0.068250115 | 22.49674906 | -1.994288968 | 0.058377996 |
| 0 - 15 | PEC | -0.09065841 | 0.06307145 | 13.61489094 | -1.437392202 | 0.17319061 |
| 0 - 15 | VC | 0.062304528 | 0.049902352 | 20.02484516 | 1.248528894 | 0.226237913 |

CD4^+^ T cell counts were log_10_-transformed. Standard error, SE; degrees of freedom, df; T statistic; t.ratio.

**Table S6:** Estimated contrasts/differences between year 15 and year 0 of CD4/CD8.

| contrast | Group | estimate | SE | df | t.ratio | p.value |
| --- | --- | --- | --- | --- | --- | --- |
| 0 - 15 | EEC | 0.01442867 | 0.089010639 | 20.57682373 | 0.16210051 | 0.872807929 |
| 0 - 15 | PEC | -0.224446533 | 0.094229589 | 17.25867757 | -2.381911401 | **0.028981294** |
| 0 - 15 | VC | 0.10625587 | 0.06753364 | 20.69055297 | 1.573376914 | 0.130799269 |

CD4/CD8 ratios were square-root transformed. Standard error, SE; degrees of freedom, df; T statistic; t.ratio.

| **Table S7:** Estimated contrasts/differences of CD4^+^ T cell counts among groups at 0, 5, 10, and 15 years of follow-up.contrast | Year | estimate | SE | df | t.ratio | p.value |
| --- | --- | --- | --- | --- | --- | --- |
| EEC - PEC | 0 | -0,094774037 | 0,095553198 | 33,04055992 | -0,991845789 | 0,587102991 |
| EEC - VC | 0 | -0,046411134 | 0,088729762 | 35,55205807 | -0,523061629 | 0,860610413 |
| PEC - VC | 0 | 0,048362903 | 0,082023686 | 31,0531388 | 0,589621191 | 0,826667892 |
| EEC - PEC | 5 | 0,012479366 | 0,070700737 | 29,33435531 | 0,176509703 | 0,982980724 |
| EEC - VC | 5 | 0,089090926 | 0,06321815 | 27,91451758 | 1,409261835 | 0,349938429 |
| PEC - VC | 5 | 0,07661156 | 0,061795683 | 30,09811382 | 1,239755855 | 0,439633436 |
| EEC - PEC | 10 | 0,027630046 | 0,062905169 | 30,38777372 | 0,439233319 | 0,899469988 |
| EEC - VC | 10 | 0,155229252 | 0,054148997 | 26,87623273 | 2,866705936 | **0,021042971** |
| PEC - VC | 10 | 0,127599206 | 0,05585399 | 31,70730276 | 2,284513709 | 0,072700183 |
| EEC - PEC | 15 | -0,049321997 | 0,067177789 | 16,9417292 | -0,734200953 | 0,746955716 |
| EEC - VC | 15 | 0,152003845 | 0,056250876 | 17,26406401 | 2,702248474 | **0,037836973** |
| PEC - VC | 15 | 0,201325842 | 0,060753426 | 17,15927505 | 3,313818743 | **0,010756916** |

CD4^+^ T cell counts were log_10_-transformed. Standard error, SE; degrees of freedom, df; T statistic; t.ratio.

**Table S8:** Estimated contrasts/differences of CD4/CD8 ratios among groups at 0, 5, 10, and 15 years of follow-up.

| contrast | Year | estimate | SE | df | t.ratio | p.value |
| --- | --- | --- | --- | --- | --- | --- |
| EEC - PEC | 0 | 0,044081863 | 0,125992222 | 31,7759013 | 0,349877654 | 0,934887322 |
| EEC - VC | 0 | 0,086206668 | 0,117353539 | 34,58863808 | 0,734589419 | 0,744803623 |
| PEC - VC | 0 | 0,042124805 | 0,108557125 | 30,73793352 | 0,388042749 | 0,920566203 |
| EEC - PEC | 5 | 0,047811088 | 0,105736984 | 28,7200909 | 0,452169958 | 0,893836728 |
| EEC - VC | 5 | 0,186361162 | 0,095030964 | 27,88395023 | 1,961057249 | 0,140792601 |
| PEC - VC | 5 | 0,138550074 | 0,09222146 | 29,21529779 | 1,502362622 | 0,304526317 |
| EEC - PEC | 10 | -0,03181398 | 0,107959922 | 29,72597181 | -0,294683242 | 0,953333808 |
| EEC - VC | 10 | 0,216970228 | 0,094020797 | 26,85529384 | 2,307683346 | 0,0718723 |
| PEC - VC | 10 | 0,248784208 | 0,095583475 | 30,98641288 | 2,602795174 | **0,036443413** |
| EEC - PEC | 15 | -0,19479334 | 0,129010593 | 25,45526803 | -1,509901903 | 0,303184984 |
| EEC - VC | 15 | 0,178033868 | 0,108849712 | 24,8963811 | 1,635593374 | 0,249847185 |
| PEC - VC | 15 | 0,372827208 | 0,115881532 | 25,49215313 | 3,217313426 | **0,009483686** |

CD4/CD8 ratios were square-root transformed. Standard error, SE; degrees of freedom, df; T statistic; t.ratio.

**Table S9:** Subgrouping EC^low^ and EC^high^ based on IL-18, IP-10 and %CD8^+^CD38^+^HLA-DR^+^.

|  | IL-18 (pg/mL) | IP-10 (pg/mL) | %CD8^+^CD38^+^HLA-DR^+^ |
| --- | --- | --- | --- |
| Median HIV-1neg (90% Percentile) | 257.10 (377.90) | 65.50 (114.10) | 1.13 (2.99) |
| EC^low^ | | | |
| PEC52 | 185.67 | 74.60 | 1.31 |
| PEC26 | 210.93 | 70.92 | 1.11 |
| PEC30 | 186.57 | 83.72 | 0.43 |
| PEC35 | 345.47 | 86.77 | 1.06 |
| EEC11 | 333.87 | 86.82 | 1.41 |
| EEC19 | 287.25 | 99.03 | 0.75 |
| EEC36 | 278.44 | 89.39 | 0.47 |
| EC^high^ | | | |
| PEC02 | 279.85 | 79.69 | 3.33 |
| PEC44 | 209.37 | 57.58 | 4.65 |
| PEC38 | 246.88 | 91.17 | 6.36 |
| PEC39 | 166.07 | 60.50 | 7.88 |
| EEC07 | 496.80 | 163.67 | 3.78 |
| EEC42 | 362.34 | 145.98 | 1.8 |
| EEC17 | 646.13 | 137.13 | 1.41 |
| EEC18 | 362.62 | 161.73 | 2.15 |

EC^low^: Elite Controllres with values of IP-10, IL-18 and %CD8^+^CD38^+^HLA-DR^+^ below the 90% percentile of HIV-1-negative individuals; EC^high^: Elite Controllres with values of IP-10, IL-18 and %CD8^+^CD38^+^HLA-DR^+^ above the 90% percentile of HIV-1-negative individuals
